# Supplementary material for: Stacking Model‐Based Classifiers for Dealing With Multiple Sets of Noisy Labels
Source: Biom J. 2025 Mar 12;67(2):e70042. doi: 10.1002/bimj.70042 (PMC11898607; doi:10.1002/bimj.70042)
Supplement: Supplementary file 1 — Supporting Information [file BIMJ-67-e70042-s001.zip › density-based-ensemble-model-main/competitors_real_data/netlab-master/netlab-master/help/glmtrain.htm]

Netlab Reference Manual glmtrain


# glmtrain

## Purpose

Specialised training of generalized linear model

## Description

`net = glmtrain(net, options, x, t)` uses
the iterative reweighted least squares (IRLS)
algorithm to set the weights in the generalized linear model structure
`net`. This is a more efficient alternative to using `glmerr`
and `glmgrad` and a non-linear optimisation routine through
`netopt`.
Note that for linear outputs, a single pass through the
algorithm is all that is required, since the error function is quadratic in
the weights. The algorithm also handles scalar `alpha` and `beta`
terms. If you want to use more complicated priors, you should use
general-purpose non-linear optimisation algorithms.

For logistic and softmax outputs, general priors can be handled, although
this requires the pseudo-inverse of the Hessian, giving up the better
conditioning and some of the speed advantage of the normal form equations.

The error function value at the final set of weights is returned
in `options(8)`.
Each row of `x` corresponds to one
input vector and each row of `t` corresponds to one target vector.

The optional parameters have the following interpretations.

`options(1)` is set to 1 to display error values during training.
If `options(1)` is set to 0,
then only warning messages are displayed. If `options(1)` is -1,
then nothing is displayed.

`options(2)` is a measure of the precision required for the value
of the weights `w` at the solution.

`options(3)` is a measure of the precision required of the objective
function at the solution. Both this and the previous condition must be
satisfied for termination.

`options(5)` is set to 1 if an approximation to the Hessian (which assumes
that all outputs are independent) is used for softmax outputs. With the default
value of 0 the exact Hessian (which is more expensive to compute) is used.

`options(14)` is the maximum number of iterations for the IRLS algorithm;
default 100.

## See Also

`glm`, `glmerr`, `glmgrad`

---

**Pages:**
Index


---

Copyright (c) Ian T Nabney (1996-9)
